# Improving the Production of Secondary Metabolites via the Application of Biogenic Zinc Oxide Nanoparticles in the Calli of *Delonix elata*: A Potential Medicinal Plant

**DOI:** 10.3390/metabo13080905

**Published:** 2023-08-02

**Authors:** Mohamed Tarroum, Norah S. Alfarraj, Fahad Al-Qurainy, Abdulrahman Al-Hashimi, Salim Khan, Mohammad Nadeem, Abdalrhaman M. Salih, Hassan O. Shaikhaldein

**Affiliations:** Department of Botany and Microbiology, College of Science, King Saud University, Riyadh 11451, Saudi Arabia

**Keywords:** *Delonix elata*, medicinal plant, tissue culture, ZnONPs, secondary metabolites

## Abstract

The implementation of nanotechnology in the field of plant tissue culture has demonstrated an interesting impact on in vitro plant growth and development. Furthermore, the plant tissue culture accompanying nanoparticles has been showed to be a reliable alternative for the biosynthesis of secondary metabolites. Herein, the effectiveness of zinc oxide nanoparticles (ZnONPs) on the growth of *Delonix elata* calli, as well as their phytochemical profiles, were investigated. *Delonix elata* seeds were collected and germinated, and then the plant species was determined based on the PCR product sequence of ITS1 and ITS4 primers. Afterward, the calli derived from *Delonix elata* seedlings were subjected to 0, 10, 20, 30, 40, and 50 mg/L of ZnONPs. The ZnONPs were biologically synthesized using the *Ricinus communis* aqueous leaf extract, which acts as a capping and reducing agent, and zinc nitrate solution. The nanostructures of the biogenic ZnONPs were confirmed using different techniques like UV–visible spectroscopy (UV), zeta potential measurement, Fourier transform infrared spectra (FTIR), X-ray diffraction (XRD), and scanning electron microscopy (SEM). Adding 30 mg/L of ZnONPs to the MS media (containing 2.5 µM 2,4-D and 1 µM BAP) resulted in the highest callus fresh weight (5.65 g) compared to the control and other ZnONP treatments. Similarly, more phenolic accumulation (358.85 µg/g DW) and flavonoid (112.88 µg/g DW) contents were achieved at 30 mg/L. Furthermore, the high-performance liquid chromatography (HPLC) analysis showed significant increments in gallic acid, quercetin, hesperidin, and rutin in all treated ZnONP calli compared to the control. On the other hand, the gas chromatography and mass spectroscopy (GC-MS) analysis of the calli extracts revealed that nine phytochemical compounds were common among all extracts. Moreover, the most predominant compound found in calli treated with 20, 30, 40, and 50 mg/L of ZnONPs was bis(2-ethylhexyl) phthalate, with percentage areas of 27.33, 38.68, 22.66, and 17.98%, respectively. The predominant compounds in the control and in calli treated with 10 mg/L of ZnONPs were octadecanoic acid, 2-propenyl ester and heptanoic acid. In conclusion, in this study, green ZnONPs exerted beneficial effects on *Delonix elata* calli and improved their production of bioactive compounds, especially at a dose of 30 mg/L.

## 1. Introduction

In this century, nanotechnology has developed into a promising field of interdisciplinary research involving the integration of biotechnology and material science. This field mainly relies on the use of ultrafine particles ranging in size from 1 to 100 nanometers, which demonstrate different properties regarding physical strength, chemical reactivity, electrical conductivity, magnetism, and photovoltaic effects compared to those of bulk materials [1]. Different approaches have been used for the fabrication of the nanoparticles, which can mostly be divided into two categories: top–down and bottom–up methods. Among bottom–up approaches, green synthesis has emerged as an eco-friendly and cost-effective method involving the use of biological materials to produce nanomaterials [2]. Green synthesis is a sustainable procedure in which the production of unwanted and harmful products is avoided. This technique is based on the use of different biological materials such as fungi, bacteria algae, and plant extracts. According to [3], the use of plant extracts is a fairly simple and easy process to produce nanoparticles on a large scale compared to bacteria- and fungi-mediated synthesis. The plant materials used in the green synthesis method of producing nanoparticles include leaf, fruit, peel, seed, and root extracts replacing the hazardous chemicals with polyphenols, flavonoids, proteins, saponins, or sugar used as the reducing and capping agent [4]. Among metal nanoparticles, zinc oxide (ZnO) is of much interest to researchers and the scientific community owing to its excellent properties in environmental sustainability. ZnONPs are biosynthesized via a very simple procedure using various plant extracts such as *Lemna minor* [5], thyme [6], *Delonix elata* [7], *Aloe vera* [8], and *Pongamia pinnata* [9]. This biosynthesis procedure does not require any additional external chemical stabilizers, as the biochemical compounds present in the plant extract can act as reducing and stabilizing agents for the synthesis [10]. Utilizing *Ricinus communis* extract seeds, ZnONPs have been well synthesized and analyzed for their efficacy regarding antioxidant, antifungal, and anticancer activities [11]. The multiplicity of plant sources used for zinc nanoparticulation has resulted in variable ZnO shapes and morphologies [10]. Emul et al. (2019) [12] noted that the morphology of the nanoparticles is very important because it is directly related to their field of use. Based on UV–visible spectroscopy and FTIR, XRD, SEM, and zeta potential analyses, nanoparticles have been characterized regarding their particle size, surface charge, and morphological and structural properties [12,13,14].

Zinc oxide nanoparticles are widely applied as an anti-microbial agent and to enhance plant growth and boost plants’ phenolic compounds [15]. A study reported by [16] showed that zinc oxide nanoparticles improved the growth of shoots and the callus of *Juniperus procera* and increased their total phenolic and flavonoid contents. In several plant species, such as *Melissa officinalis* [17], *Capsicum annuum* [15], *Raphanus sativus* L. [18], and *Linum usitatissimum* L. [19], ZnONPs have been shown to trigger the production of secondary metabolites. Zinc oxide nanoparticles improve plant growth and metabolism via different mechanisms such as the improvement of nutritional status, the modification of enzymatic and non-enzymatic antioxidant systems, the altering of phytohormonal balances, their influence on nitrogen assimilation, and by affecting gene expression [17]. In this context, García-López et al. (2018) [15] reported that ZnO can modify the level of auxin via tryptophan biosynthesis. Depending on the concentration used, nanoparticles may have beneficial or harmful effects. Indeed, a high level of nanoparticles leads to severe stress, which can promote the production of stress-induced secondary metabolites [20].

*Delonix elata* is a tree found in the southern region of Saudi Arabia. This species belongs to the Fabaceae family and the Caesalpinioideae sub-family [21]. The *Delonix* genera consist of three species, two of which are found in eastern Africa. In Greek, the name *Delonix* is derived from Delos, meaning a claw that looks like petals, and elata, meaning tall. *D. elata* is commonly named the creamy peacock flower, the flamboyant tree, the tiger bean, or the white gul mohur [22]. *D. elata* was used in folk medicine to treat joint pain and flatulence. It has been noted that the local people in some areas use the leaves and bark of *D. elata* to treat inflammation. In [23], the anti-inflammatory activity of *D. elata* leaves and bark extracts was found to be significant. The antibacterial activity of the *D. elata* leaf extract was reported, and this was attributed to the presence of bioactive compounds such as luteolin [24]. Phytochemical analysis using gas chromatography–mass spectrometry (GC-MS) showed that the *D. elata* methanolic extract mainly contains flavonoids and terpenes [25]. On the other hand, the HPLC analysis of the *D. elata* ethanolic extract revealed the presence of antioxidant molecules such as gallic acid, ellagic acid, coumaric acid, quercetin, and rutin [26].

The in vitro culture can represent a good alternative to cultivation in the field and facilitate the production of metabolites that have a commercial interest [27]. In addition, plant tissue culture accompanying nanoparticles has been showed to be a reliable alternative for the induction of bioactive compounds in medicinal plants [28]. In a previous study, the leaves, stem, and roots of *D. elata* have been used as explants to induce callus [29]. However, no studies showed the effects of nanoparticles generally and, especially, ZnONPs on the callus growth as well as the biosynthesis of secondary metabolites in *D. elata*. Thus, in this study, we aimed to obtain green ZnONPs utilizing the leaf extract of *Ricinus communis*. In addition, we tested the effects of the ZnONPs on the phytochemical profile of the callus induced by the potent medicinal plant *Delonix elata*.

## 2. Materials and Methods

### 2.1. Zinc Oxide Nanoparticles Biosynthesis and Characterization

The green synthesis of ZnONPs was performed by following the protocol described by [30]. Briefly, 15 g powder of the *Ricinus communis* leaves (voucher specimen KSU 13719 deposited at the Herbarium, College of Sciences, King Saud University, KSA) was soaked in 300 mL of deionized Milli-Q water. After heating in the water bath (Julabo TW20, Seelbach, Germany) at 60 °C for 30 min, the mixture was kept shaking overnight at 37 °C using a New Brunswick Innova^®^ 44 machine. Thereafter, the extract was filtered through Whatman filter paper no. 1. To 200 mL of the obtained filtrate, 3.78 g of zinc nitrate hexahydrate (Zn(NO_3_)_2_·6H_2_O) was added and placed at hot plate magnetic stirrer for 2 h at 60 °C (Janke & Kunkel, Staufen, Germany). The mixture was centrifuged at 10,000 rpm for 10 min at 4 °C, the supernatant was discarded, and the precipitates were washed several times. The precipitates were then dried at 50 °C in a hot air oven (Vaciotem-T oven, JP Selecta SA, Barcelona, Spain) and finally calcinated at 500 °C for 3 h in a furnace (DKN 602, Yamato Scientific Co., Ltd., Tokyo, Japan).

The ZnONPs were characterized via different techniques such as UV–visible (UV-vis), Fourier transform infrared spectra (FTIR), X-ray diffraction (XRD), scanning electron microscope (SEM), and zeta potential. The UV-vis was performed using the spectrophotometer instrument (Shimadzu UV-1800, Chiyoda-ku, Tokyo, Japan) in the range of 300 to 700 nm. As to the FTIR, a Fourier transform infrared spectrophotometer (8400S, Shimadzu, Tokyo, Japan) was employed to determine the functional groups in plant extract and ZnONP solution in the spectral array from 400 to 4000 cm^−1^. The XRD was measured in the Bruker D8 Discover XRD instrument at 2θ ranging from 10 to 80°. Further, to determine the particle’s shapes and sizes, a scanning electron microscope (SEM) was used. The surface charge or the zeta potential of the ZnONPs was identified using Malvern Zetasizer machine (Malvern Instruments Ltd., Malvern, UK).

### 2.2. Plant Seeds Collection and Germination

The seeds of the species *Delonix elata* were collected from the Baljurashi, Al-Baha region, Saudi Arabia. The seeds were placed in an airtight container containing silica gel and delivered to the plant biotechnology lab in the College of Science, King Saud University. In the lab, the seeds were sterilized for 10 min in 50% (*v*/*v*) commercial Clorox bleach and, thereafter, washed several times with sterile distilled water. Afterward, the seeds were sown in jars containing 1/4 strength MS media supplemented with 2% sucrose and solidified 0.5% of agar. The jars were maintained in a growth chamber at 25–28 °C under 16 h/8 h photoperiod/dark cycle for three weeks. Once the germination was achieved, the germinated seedlings were used to identify the plant species via a molecular-based method and to initiate the callus formation.

### 2.3. Molecular Identification

The isolation of the DNA from the germinated seedlings was performed using the DNeasy Plant Mini Kit (Qiagen, Hilden, Germany), as described in the manufacturer’s instructions. The DNA quantity and integrity were checked in Nanodrop 8000 spectrophotometer (Thermo Scientific, Wilmington, NC, USA) and 1% agarose gel electrophoresis, respectively. Thereafter, the amplification of the ITS region was accomplished in a total volume of 25 µL, which consists of PuReTaqTM Ready-To-GoTM PCR beads (GE Healthcare, Little Chalfont, Buckinghamshire, UK), 0.5 µL of forward primer (ITS1), 0.5 µL of reverse primer (ITS4), 2 µL of DNA, and 20 µL of ultra-pure water. The PCR amplification was achieved in Applied Biosystems thermal cycler (Applied Biosystems, Waltham, MA, USA) following the conditions cited by [31], including one cycle initial denaturation at 94 °C for 5 min, then denaturation at 94 °C for 1 min, annealing at 50 °C for 1 min, extension at 72 °C for 1 min for 30 cycles, and a final extension step of 72 °C for 5 min. A total of 5 µL of the PCR product was checked in 1.2% agarose gel and then bidirectionally sequenced in the Macrogen Inc. (Geumchun-gu, Seoul, Republic of Korea). The identification of the plant species was performed via the blast of the obtained sequence in the NCBI databases.

### 2.4. Callus Induction and ZnONP Application

The germinated seedlings were employed as explants to induce callus. Around 1 cm of the donor seedlings was transferred to MS media [32], amended with 30 g/L sucrose, and enriched with different combinations (0.5, 1, 2.5, and 5 µM) of 2,4-dichlorophenoxyacetic acid (2,4-D) and 6-Benzylaminopurine (BAP). Jars were kept in the growth chamber at controlled condition for six weeks, and the best combination (2.5 µM 2,4-D and 1 µM BAP) for callus induction was selected. Subsequently, the effects of the ZnONPs on callus growth were tested by transferring the obtained callus onto agar media containing MS mineral media, 30 g/L sucrose, 2.5 µM 2,4-D, 1 µM BAP, and supplemented with 0, 10, 20, 30, 40, and 50 mg/L of ZnONPs. Six weeks later, the callus fresh weights were recorded and then dried to extract the phytochemical compounds.

### 2.5. Phytochemicals Extraction

The callus of the different treatments was dried at room temperature and then powdered in the TissueLyser LT machine (Qiagen, Hilden, Germany) instrument. A total of 2 g of the powder was soaked in 20 mL methanol (80%) and kept shaking for 24 h. Next, the mixtures were centrifuged for 5 min at 5000 rpm, and the supernatants were collected and concentrated via evaporation. The obtained extracts were finally dissolved in 2 mL of methanol, filtered using a syringe filter of 0.45 μm, and kept at 4 °C for further analysis.

#### 2.5.1. Total Phenolic Content Estimation

The Folin–Ciocalteu colorimetric method described by [33] was employed to estimate the total phenolic content. For that, 0.5 mL of the diluted (1:5) callus extract was mixed with 125 μL of Folin−Ciocalteu and then allowed to react for 5 min. Thereafter, 1250 μL of sodium carbonate (7%) and 1125 μL of water were added. The mixture was incubated at room temperature for 90 min, and then the absorbance of the reaction was measured at 760 nm using UV-1800 spectrophotometer (Shimadzu UV spectrophotometer). The values of the total phenolic content were compared to the calibration curve prepared with known doses of gallic acid and expressed as microgram gallic acid per gram dry weight.

#### 2.5.2. Total Flavonoid Content Estimation

The total flavonoids of the callus extracts were determined using the calorimetric method described by [34]. The reaction mixture containing 0.5 mL of the sample extract and 0.5 mL of 2% AlCl_3_ was incubated for 60 min at room temperature. Afterward, the absorbance of this mixture was spectrophotometrically measured at 420 nm. The values of the total flavonoid contents were expressed as micrograms of quercetin per gram dry weight.

#### 2.5.3. HPLC Quantification

The HPLC quantification of the gallic acid, quercetin, hesperidin, and rutin was performed using an Agilent Technologies 1290 Infinity instrument equipped with ZOBRAX RX-C18 column (4.6 × 150 mm), which was used for the separation of the target compounds as follows:

The gallic acid in the treated callus extracts was detected using 60% methanol and 40% acetic acid as a mobile phase, which was pumped at a flow rate of 1 mL/min, with a 5 min run time and 1 µL of injection volume at 25 °C and at a wavelength of 274 nm.

For the quercetin quantification, the mobile phase was composed of methanol and acetonitrile in a ratio of 60:40. The injection volume was 1 µL, the flow rate was 1 mL/min, the run time was 5 min, and the DAD detector was set at 254 nm.

As for the Hesperidin separation and quantification, the mobile phase consisted of 80% methanol and 60% acetonitrile, the flow rate was 0.5 mL/min, the injection volume was 1 µL, the column temperature was set at 25 °C and DAD detection at 280 nm.

Regarding the rutin separation and quantification, column temperature was maintained at 25 °C, the mobile phase consisted of methanol and acetonitrile (80%:20%), the flow rate was 0.8 mL/min, the run time was 5 min, and the DAD (UV-vis) detector was set at 254 nm.

The targeted compounds in the callus extracts were calculated by referencing them to the calibration curves generated from the Area (mAU*s) of the authentic standards giving an R^2^ equal to 0.989, 0.990, 0.999, and 0.987, respectively, for gallic acid, quercetin, hesperidin, and rutin.

#### 2.5.4. Callus Extracts GC-MS Analysis

The extracts of the treated and untreated callus were subjected to gas chromatography–mass spectrometry (GC-MS) analysis using QP2010 Ultra, Shimadzu, Tokyo, Japan instrument fitted with an Rtx-5MS column (30 m; 0.25 mm inner diameter; 0.25 µm). The helium gas was used as the carrier gas at a constant flow rate of 1.6 mL/min. The GC oven temperature program was set as follows: 50 °C for 3 min, increased at 10 °C/min to 280 °C, held for 3 min, elevated to 300 °C at 2 °C/min, and then maintained at this temperature for 10 min. The injector and detector temperatures were programmed to hold 250 and 275 °C, respectively. For the mass spectrometer, the electron ionization energy was set at 70 eV. The phytochemicals of the callus extracts were identified by comparing their mass spectra and retention time to those stored in the National Institute of Standards and Technology (NIST) database. 

The leaf extract of *Ricinus communis* was used as a stabilizing and reducing agent in the synthesis of ZnONPs. The extract was filtered using a syringe filter (0.45 µm) and then subjected to the GC-MS analysis, as described above.

### 2.6. Statistical Analysis

The statistical analysis was carried out using IBM SPSS Statistics, version 25 (SPSS Inc./IBM Group, Armonk, NY, USA). The treatments were made in triplicates, and their data were subjected to one-way ANOVA followed by an analysis with Duncan’s test. The different letters on chart bars presented a significant difference at *p* ≤ 0.05.

While for plant species identification, the NCBI-BLAST search was performed, and then the MEGA software package (version 11, http://www.megasoftware.net), was used to construct the phylogenetic tree.

## 3. Results

### 3.1. Biosynthesis and Characterization of the ZnONPs

**Figure 1:**
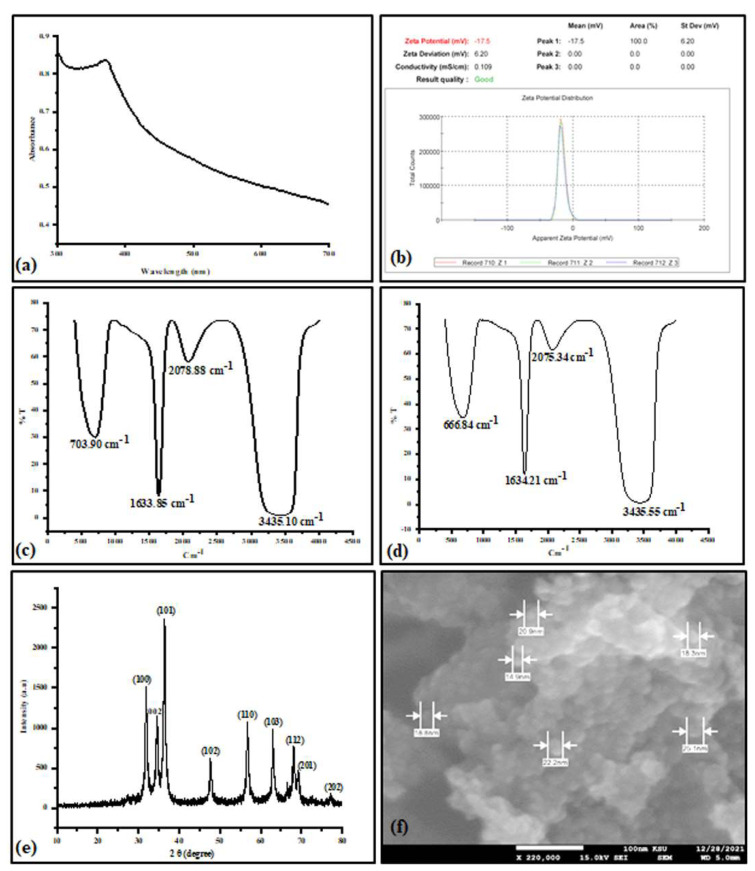
Zinc oxide nanoparticle (ZnONP) characterization: UV−visible spectra (**a**), zeta potential (**b**). Fourier transform infrared spectroscopy (FTIR) of the plant extract (**c**), FTIR of the synthesized ZnONPs (**d**), XRD pattern of ZnONPs (**e**), and scanning electron microscope (SEM) (**f**).

The ZnONPs were biologically prepared using the aqueous extract of *Ricinus communis*, which was analyzed for its phytoconstituents using GC-MS. This analysis showed the existence of 15 compounds, where silanediol dimethyl (5.25%), 2-Methoxy-4-vinylphenol (5.69%), and ricinine (65.36%) were the main compounds identified based on the area percentage (Table 1). These three compounds categorized, respectively, as flavonoid, phenol, and alkaloid, might play an important role in the capping and stabilizing of nanoparticles during biosynthesis. The produced ZnONPs were firstly characterized by UV–visible spectrophotometer at the optical spectrum ranging from 300 to 700 nm. A strong peak at around 370 nm was observed confirming the presence of ZnONPs in the mixture (Figure 1a). The colloidal solution stability was determined using the zeta potential test based on the surface charge of the fabricated nanoparticles. This test showed that the ZnONP zeta potential value was −17.5 mV, which indicates that the produced nanoparticles were negatively charged and highly stable (Figure 1b). The detection of the functional groups in the plant extract and the ZnONP colloidal solution was performed using Fourier transform infrared spectroscopy in the wavenumber ranging from 500 to 4500 cm^−1^. The plant extract (Figure 1c) showed four peaks at 709.90, 1633.85, 2078.88, and 3435.10 cm^−1^, which were, respectively, shifted to 666.84, 1634.21, 2075.34, and 3435.55 cm^−1^ in the ZnONP solution (Figure 1d). The medium peaks (709.90 and 666.84 cm^−1^) correspond to C-H bending, the strong peaks (1633.85 and 1634.21) are attributed to C=O stretching, the weak peaks (2078.88 and 2075.34 cm^−1^) correspond to C≡C stretching, and the other strong peaks (3435.15 and 3435.55 cm^−1^) are assigned to O-H stretching. The XRD pattern of the fabricated ZnONPs is presented in Figure 1e. Strong and sharp peaks were observed at 2θ values equal to 31.84, 34.62, 36.42, 47.71, 56.78, 63.04, 68.06, 69.42, and 77.18. Compared to JCPSD card no. 01-007-2551, these peaks, respectively, correspond to (100), (002), (101), (102), (110), (103), (112), (201), and (202) diffraction planes. Further, using Scherrer’s formula, the average crystallite size of the ZnONPs was to be 13.5 nm. According to the SEM analysis (Figure 1f), the nanoparticles are agglomerated showing a spherical morphology and having sizes varied from 14.9 to 25.1 nm.

### 3.2. Delonix elata Molecular Identification

**Figure 2:**
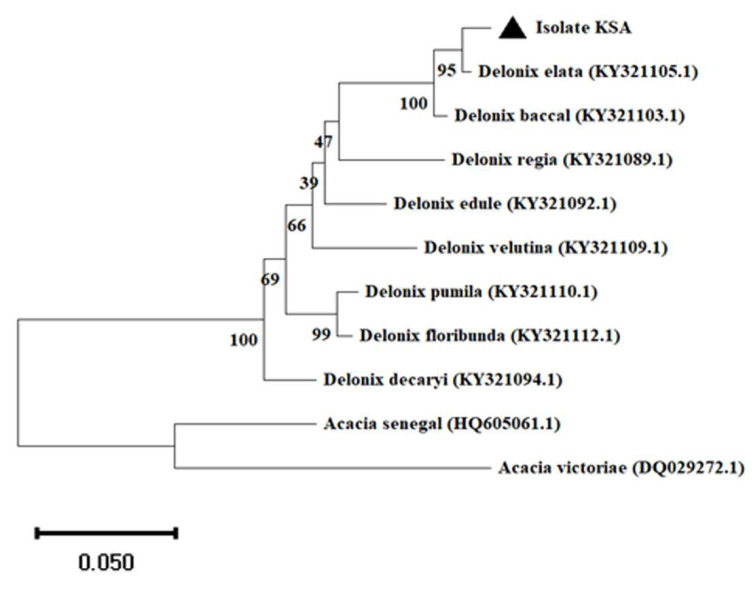
Identification of *Delonix elata* via amplification of its internal transcribed spacer (ITS) region. Neighbor-joining method in MEGA X software was used to construct the phylogenetic tree. Scale bar: 0.05 substitutions per nucleotide.

The species of the collected plant was identified with the amplification of the internal transcribed spacer (ITS) as we described in the Materials and Methods section. Blasting the assembled sequences to the NCBI GenBank, we noted that the highest identity (98.77%) was with *D. elata* published under the accession number KY321105.1. Further, the constructed phylogenetic tree based on the neighbor-joining method revealed that the collected plant was associated with *D. elatata*. The phylogenetic tree was rooted in *Acacia senegal* and *Acacia victoriae*, which were selected as an outgroup to the group containing *Delonix* species (Figure 2).

### 3.3. Effect of ZnONPs on Callus Growth

**Figure 3:**
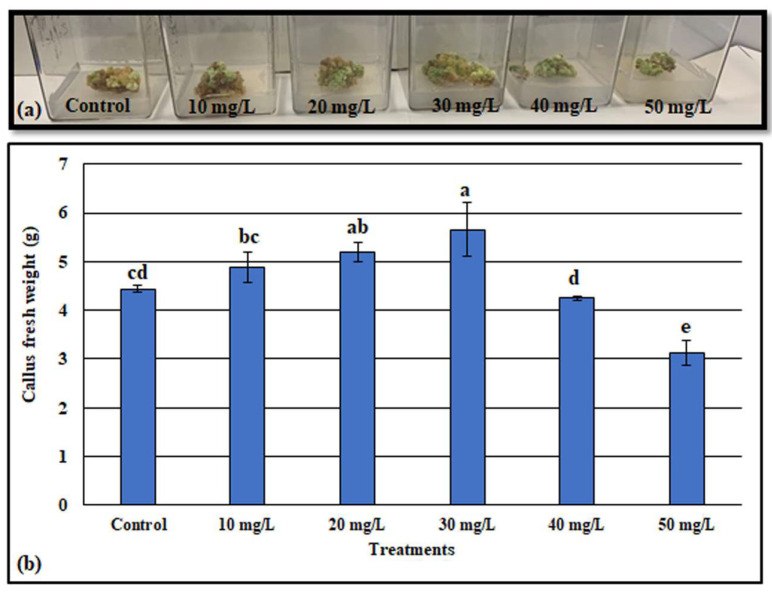
Effect of various concentrations of ZnONPs on *D. elata* callus morphology (**a**), callus fresh weight (**b**). Values are the means of three replicates ± SD; the significant differences according to Duncan’s test (*p* < 0.05) were indicated by the different letters on the bars.

The calli derived from the *D. elata* explants were inoculated with 0, 10, 20, 30, 40, and 50 mg/L of ZnONPs as we described in the Materials and Methods section. The callus morphological aspects and their fresh weights are shown in Figure 3. After six weeks of inoculation with the different concentrations of ZnONPs, we observed that the callus fresh weight varied depending on applied doses. Indeed, the callus with the highest fresh weight (5.65 g) was recorded in the culture medium supplemented with 30 mg/L of ZnONPs. However, the lowest callus fresh weight was observed in the medium containing 50 mg/L of ZnONPs. Additionally, we noted that when the ZnONP doses were increased further than 30 mg/L, there was a decrease in the callus growth.

### 3.4. Phytochemical Analysis

#### 3.4.1. Effects of ZnONPs on Phenols and Flavonoids Contents

**Figure 4:**
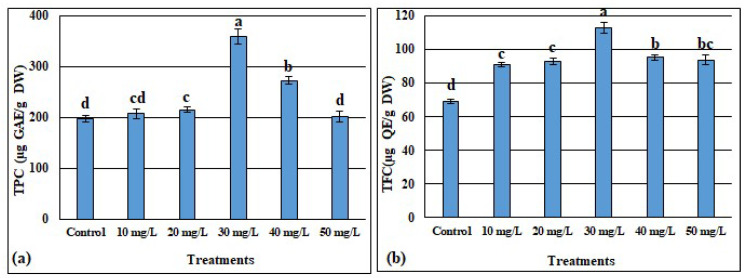
Effect of different concentrations of ZnONPs on total phenolic content expressed as gallic acid equivalent (**a**) and total flavonoid content expressed as quercetin equivalent (**b**) in the *Delonix elata* callus. Values are the means of three replicates ± SD; the significant differences according to Duncan’s test (*p* < 0.05) were indicated by the different letters on the bars.

The total phenolic content (TPC) of the treated callus is shown in Figure 4a. We observed that the highest TPC (358.85 µg GAE/g of DW) was produced by the callus cultivated in the media supplemented with 30 mg/L ZnONPs, while, when the culture media was not provided with ZnONPs, the lowest TPC (201.45 µg GAE/g of DW) was recorded. Generally, compared to the control, the TPC was increased by 1.05, 1.09, 1.82, 1.38, and 1.02 times, respectively, for the callus grown with 10, 20, 30, 40, and 50 mg/L of ZnONPs.

Similarly, for the total flavonoids content (TFC), the highest value (112.88 µg QE/g DW) was recorded in the calli treated with the 30 mg/L of ZnONPs, whereas the lowest amount of TFC (69.14 µg QE/g DW) was registered in the calli of the media without ZnONPs. Compared to the control, the TFC was significantly improved by 31.6%, 34.33%, 63.26%, 37.7%, and 35.49% following the exposure to 10, 20, 30, 40 and 50 mg/L, respectively. Further, even though there was a decrease in TFC when the concentrations of ZnONPs were greater than 30 mg/L, the amount of TFC remained greater than those detected at doses below 30 mg/L of ZnONPs (Figure 4b).

**Figure 5:**
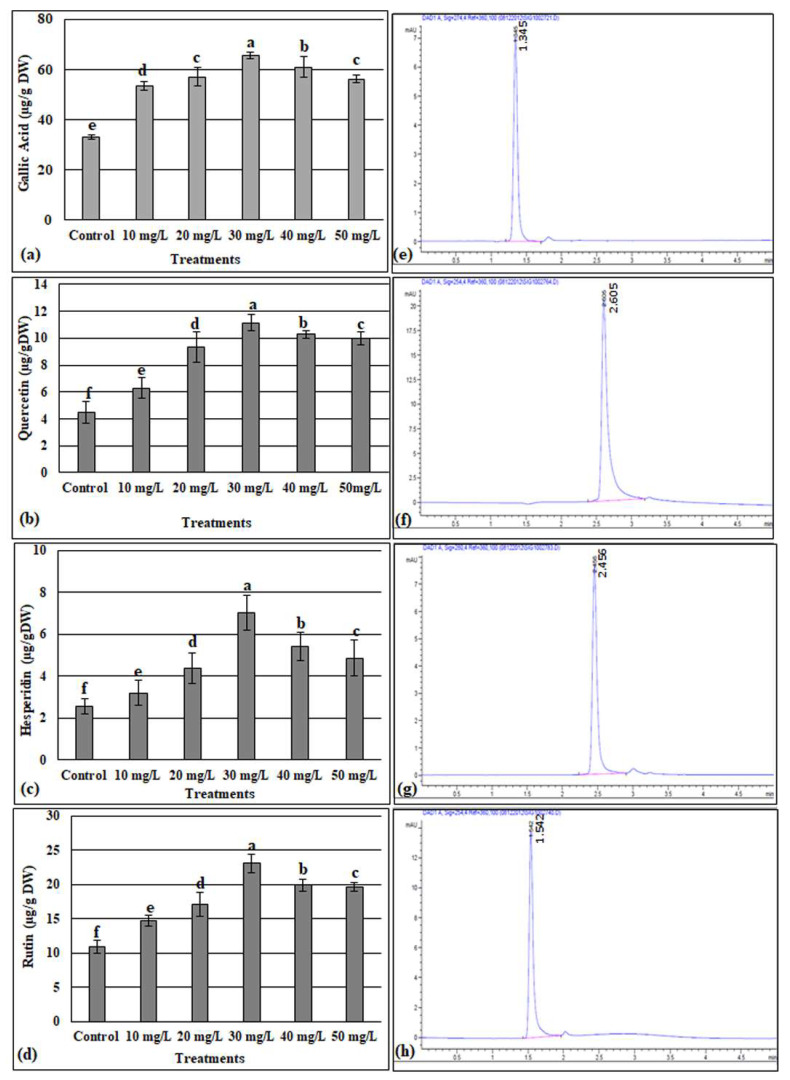
HPLC quantification of gallic acid (**a**). Quercetin (**b**), hesperidin (**c**), and rutin (**d**) in the ZnONP-treated and -untreated callus of *D. elata*. (**e**–**h**), respectively, presented the chromatograms of the standards of gallic acid, quercetin, hesperidin, and rutin. Values are the means of three replicates ± SD; the significant differences according to Duncan’s test (*p* < 0.05) were indicated by the different letters on the bars.

On the other hand, one phenolic compound (gallic acid) and three flavonoids (quercetin, hesperidin, and rutin) of the treated and untreated callus were quantified using HPLC (Figure 5). The identification of these compounds in the callus extracts was performed by matching their retention times to those of their specific standards under similar conditions. The retention times of gallic acid, quercetin, hesperidin, and rutin were found to be 1.345, 2.05, 2.456, and 1.542 min, respectively (Figure 5e–h). This study showed that all tested compounds, gallic acid (Figure 5a), quercetin (Figure 5b), hesperidin (Figure 5c), and rutin (Figure 5d) were significantly increased in the callus supplemented with all doses of ZnONPs compared to untreated callus. Further, among the ZnONP-treated callus, the highest contents of gallic acid, quercetin, hesperidin, and rutin (65.45, 11.12, 7.03, and 23.07 µg/mg, respectively) were detected in the callus treated with 30 mg/L of ZnONPs.

#### 3.4.2. GC-MS for Phytochemical Compound Identification

**Figure 6:**
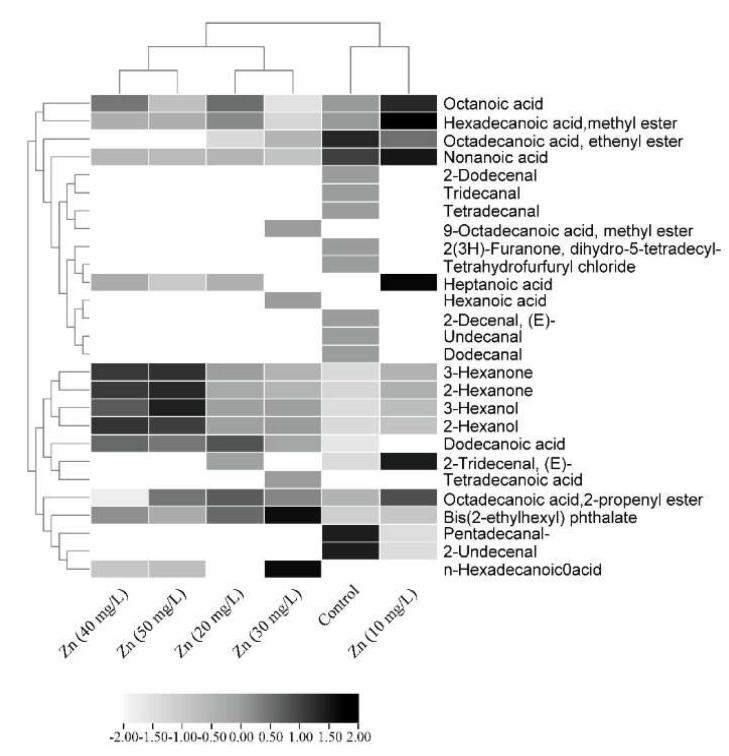
Heatmap showing the phytochemicals compounds among calli treated with ZnONPs. The color level in the key represents the abundance of the compounds basing GC-MS area percentage.

Gas chromatography and mass spectroscopy (GC-MS) was used to detect the bioactive compounds in the extracts of the calli treated and untreated with ZnONPs. The result of this test revealed 20, 14, 13, 15, 12, and 12 phytochemical compounds, respectively, identified in the callus extracts treated with 0, 10, 20, 30, 40, and 50 mg/L of ZnONPs. Based on the area percentage, the highest compounds detected in the extracts were octadecanoic acid, 2-propenyl ester (10.04%) for the control callus, and heptanoic acid and octadecanoic acid, 2-propenyl ester (13.37% and 31.31%, respectively) for the callus treated with 10 mg/L of ZnONPs, while the bis (2-ethylhexyl) phthalate was the major compound detected in the extracts of the callus treated with 20, 30, 40, and 50 mg/L of ZnONPs, where their area percentages were 27.33%, 38.68%, 22.66%, and 17.98%, respectively. Overall, the retention time, the area percentage, and the biological activities of the annotated phytocompounds are shown in Table 2. Further, the heatmap showing the hierarchical clustering of the identified phytocompound as well as of the ZnONP treatments is presented in Figure 6.

## 4. Discussion

Nanotechnology is a procedure of the synthesis, characterization, and manipulation of nanoparticles at sizes in the nanoscale ranging from 1 to 100 nm. Nanotechnology as a multidisciplinary field impacts several domains, such as engineering, biology, chemistry, computing, materials science, communications, etc. [60]. For instance, in the agriculture field, nanoparticles have been introduced as nanofertilizers for plant growth and protection, as nanobiosensors for the detection of plant diseases and biological components and also for genetic engineering and gene editing [61]. The use of expensive and toxic materials for the fabrication of nanoparticles has become a major concern. Thereby, many researchers have resorted to using environmentally friendly bio-renewable materials like vegetable oils, carbohydrates, and plant extracts [62]. Plant extracts contain biomolecules that can be used for reducing metal ions to nanoparticles in a biogenic process. This reduction of metal ions to nanoparticles is a rapid procedure, environmentally friendly, performed at room temperature and pressure, and scaled up easily. Water-soluble metabolites, such as alkaloids, phenolic compounds, and terpenoids, and coenzymes are the major reducing agents involved in the nanoparticle’s green synthesis [63]. The extracts of several plant species were successfully employed in producing nanoparticles [64,65,66,67]. Nanoparticles, in turn, have been used as elicitors to improve the plant’s bioactive compounds, as these compounds are commercially valued to be widely used in the medical, pharmacological, cosmetic, agriculture, and food industries [68].

In this current study, ZnONPs were biologically synthesized and investigated for their effects on phytochemical compounds in the callus derived from the seedlings of *D*. *elata*. The Delonix species was first identified via the amplification of the internal transcribed spacer (ITS) region, as this region was commonly used for plant molecular systematic investigations at the species level [31].

For the fabrication of ZnONPs, the leaves extract of *Ricinus communis* L. containing ricinine (65.36%), silanediol dimethyl (5.25%), and 2-Methoxy-4-vinylphenol (5.69%) as the major chemical constituents (Table 1), was used as capping and reducing agents. Chennimalai et al. [69] reported that ZnONPs were synthesized via a facile green approach from the *Ricinus communis* L. leaf extract. This facile approach was attributed to the presence of the secondary metabolites, which were qualitatively examined using the standard method of screening. The plant-based bioconstituents such as alkaloids, flavonoids, and phenolics play the role of strong chelating, reducing, and stabilizing agents, which ensure stability, prevent agglomeration, and help to adjust the shape and size of the nanoparticles [70]. The green synthesis of ZnONPs was first confirmed via UV–visible spectroscopic analysis, which showed an absorption peak at 370 nm (Figure 1a). The absorption peak at this range could be explained by the intrinsic band-gap absorption of ZnO, possibly from the electron exchange from the valence band to the conduction band [69,71]. The ZnONP colloidal solution stability was assessed using the zeta potential based on the charge of fabricated nanoparticles [72]. Jan et al. (2021) [73] reported that the negative charge on the particle’s surface is effective for particle dispersion stability. It also prevents nanoparticle aggregation, which can happen because of the high binding affinity between the extracts and the metallic ion. The zeta potential analysis showed a value of −17.5 mV (Figure 1b), indicating the stability of the produced nanoparticles. Almost similar results of zeta potential (−18.4 mV) were observed when ZnONPs were fabricated using the aqueous leaf extract of *Aquilegia pubiflora* [73]. On the other hand, the FTIR analysis showed almost the same dips when comparing the absorption peaks detected in the plant extract and those of the ZnONP solution (Figure 1c,d). This confirmed the presence of the plant extract secondary metabolites on the surface of the nanoparticles playing the roles of capping and stabilizing agents. Further, the absorption peaks detected at (3435.15 and 3435.55 cm^−1^), (2078.88 and 2075.34 cm^−1^), (1633.85 and 1634.21 cm^−1^), and (709.90 and 666.84 cm^−1^) are, respectively, ascribed to alcohol OH, alkynes, carboxylic acids, and aromatic compounds as functional groups [74,75,76]. According to [77], the slight shifting peaks between the plant extract and the synthesized nanoparticles are owing to the decrease in the value of the force constant and the increase in the reduced mass, which can simultaneously act to provoke a shift in the FTIR peaks. The ZnONPs were characterized also for their crystalline nature, crystal morphology, and size using XRD and SEM techniques (Figure 1e,f). The analysis of XRD peaks and their corresponding hkl planes revealed the crystalline nature with the wurtzite hexagonal shape of the produced nanoparticles according to JCPDS card no. 36-1451. Furthermore, the SEM analysis displayed spherical nanoparticles with an average size of 20 nm, confirming the potential of the *Ricinus communis* L. leaf extract for the fabrication of the nanoparticles [78,79].

Nanoparticles were used for a wide purpose. In the in vitro culture, the nanoparticles were successfully employed to eliminate microbial contamination from the culture media. Further, the nanoparticle application was found to be effective for callus induction, somatic embryogenesis, organogenesis somaclonal variation, genetic transformation, and secondary metabolite production [80,81]. It is possible to conquer the recalcitrant potential of cultivars to adventitious organogenesis when the culture medium is enriched by the zinc nanoparticles [82]. Alharby et al. (2017) [83] observed that the inclusion of ZnONPs in culture media stimulated the callus growth of *Solanum lycopersicum*, and the dose of 30 mg/L was found to be more suitable for growth enhancement. Likewise, in this study, the highest *D. elata* callus fresh weight (5.65 g) was obtained in the media supplemented with 30 mg/L of ZnONPs (Figure 3), while above 30 mg/L the higher the concentration of ZnONPs, the lower callus fresh weight. The positive role of ZnONPs in callus growth improvement may be explained by the involvement of the Zn in protein synthesis, carbohydrates, lipids, and nucleic acids metabolism [84]. Moreover, Zinc may act as a biosynthesis stimulator of plant growth regulators, especially the auxins and gibberellins [20,85]. Similar to our findings, [30] noted that when the dose of ZnONPs was more than 30 mg/L, the callus induction frequency of *Panicum virgatum* L declined. The negative effects of ZnONPs may be due to the injury in the cell’s wall and membrane after high dose application. In this regard, [86] noted that at high concentrations of ZnONPs and over time, the nanoparticles can aggregate into micro-size particles, which reduce their dissolution, and, thus, be similar in pattern to the micro-form.

Besides having an organogenesis fate, callus also has the potential for the same chemical as the plant and can, therefore, be used for secondary metabolites production [76]. The in vitro regeneration of medicinal plants is used by industrial companies to produce bioactive compounds in a relatively short period of time for ensuring continuous supply [87]. In the presence of ZnONPs, the total phenol and total flavonoid contents were increased depending on the applied ZnONP doses reaching the maximum values (358.85 µg GAE/g DW and 112.88 µg QE/g DW, respectively, for the total phenols and total flavonoids) at 30 mg/L and then decreased at above this dose (Figure 4). A similar trend was recorded by [20], where the maximum values of total phenols and total flavonoids were registered at 25 mg/L of ZnONPs for the calli derived from the stem of *Linum usitatissimum* L. For the callus cultures of *Silybum marianum* L., it was also observed that ZnONPs significantly enhanced the phenolic and flavonoid contents, and the highest values were recorded in the cultures containing 0.15 mg/L of ZnONPs [88]. According to [89], the use of nanoparticles leads to the synthesis of secondary metabolites such as phenols and flavonoids, which play the role of ROS scavengers. Owing to their small nano size, ZnONPs having the high potential to move into the cells via apoplastic or symplastic routes, could activate the synthesis pathways of the polyphenols and enhance their accumulation in the cell [88]. Identically to the trend showed for the total phenol and total flavonoids following the application of ZnONPs, the quantification via HPLC showed that the highest values of gallic acid, quercetin, hesperidin, and rutin were detected in the calli treated with 30 mg/L of ZnONPs, before declining at 40 and 50 mg/L of ZnONPs (Figure 5). In agreement with our findings, [90] noted that Fe-ZnO-NPs improved the biomass and activate the secondary metabolism in cell cultures of *Fagonia indica.* García-López et al. [15] documented that zinc oxide nanoparticles boosted the phenolic compounds of *Capsicum annuum* L. It was speculated that this might be because of the changes induced by ZnONPs in the physiology and biochemistry of plants, which lead to the synthesis of the secondary metabolites. The phenols and flavonoids are potent hydrogen donors responsible for several biological activities owing to their carboxyl and hydroxyl functional groups [91].

The total phytochemical compounds in the treated and untreated callus extracts were screened using GC-MS. The detected phytoconstituents as well as their biological activities were reported in Table 2. The analysis based on the area percentage peaks showed that octadecanoic acid, 2-propenyl ester with antibacterial activity [55] was the predominant compound for the control callus extract. For the 10 mg/L ZnONP-treated callus, the two compounds, heptanoic acid and octadecanoic acid, 2-propenyl ester, showed almost the same peak area of 13.37 and 13.31%, respectively, while bis(2-ethylhexyl) phthalate possessing antibacterial and antimutagenic activities was the most dominant compound in 20, 30, 40, and 50 mg/L ZnO-NPs calli extracts with the peak area of 20.33, 38.68, 22.66, and 17.98%, respectively [58,59]. In a previous study performed on *Delonix elata*, the GC-MS analysis identified 50 and 45 compounds, respectively, in the roots and leaves extracts. Among these detected compounds, quercetin, hesperidin, and rutin had the highest percentage peaks [25]. These phytochemical compounds presented in *Delonix elata* extracts may support its use as a potent medicinal plant [22].

## 5. Conclusions

In conclusion, the fabrication of ZnONPs using the aqueous leaf extract of *Ricinus communis* was confirmed via UV-vis absorption, zeta potential, FTIR, XRD, and SEM techniques. The addition of ZnONPs up to 30 mg/L significantly increased the *Delonix elata* callus development. However, above 30 mg/L, the higher the concentration of ZnONPs, the lower the callus fresh weight. On the other hand, all doses of ZnO NPs (10, 20, 30, 40, and 50 mg/L) improved the accumulation of the phenolic and flavonoid compounds. This was clearly remarked in the HPLC quantified gallic acid, quercetin, hesperidin, and rutin compounds. Further, the GC-MS analysis revealed that a higher number of compounds (20) was detected in the extract of the control callus. However, fewer compound numbers (12) were detected in 40 and 50 mg/L ZnONP-treated calli. The main constituent in the control callus is octadecanoic acid, 2-propenyl ester. The extract of 10 mg/L ZnONPs mainly contains octadecanoic acid, 2-propenyl ester and heptanoic acid (13.37 and 13.31%, respectively), whereas the 20, 30, 40, and 50 mg/L ZnONPs calli extracts were mainly rich on bis (2-ethylhexyl) phthalate. Therefore, the difference in phytochemical compounds among the treated and untreated calli should be conducted for further investigations to understand the mechanism behind it.

## Figures and Tables

**Table 1:** GC-MS analysis of *Ricinus communis* leaf extract.

NO	RT (min)	%	Compounds
1	5.267	5.25	Silanediol, dimethyl-
2	6.989	1.34	2-Furanmethanol
3	8.156	1.57	1,2-Cyclopentanedione
4	8.34	2.37	Undecane
5	8.391	2.46	Dodecanoic acid, 1,1-dimethylpropyl ester
6	9.118	2.36	Cyclopentasiloxane, decamethyl-
7	9.192	1.08	2(5H)-Furanone
8	9.936	2.70	Benzeneacetaldehyde
9	11.601	2.96	2-Propen-1-ol
10	11.984	0.93	p-Cresol
11	12.442	2.69	4H-Pyran-4-one, 2,3-dihydro-3,5-dihydroxy-6-methyl-
12	14.971	5.69	2-Methoxy-4-vinylphenol
13	15.601	1.96	5-Hydroxymethylfurfural
14	19.446	1.27	p-Hydroxyphenylacetone
15	28.738	65.36	Ricinine

**Table 2:** Phytochemical compounds detected in ZnONP-treated and -untreated calli extracts of Delonix elata.

Compounds	Control		Zn (10 mg/L)		Zn (20 mg/L)		Zn (30 mg/L)		Zn (40 mg/L)		Zn (50 mg/L)		Biological Activity
	RT	%	RT	%	RT	%	RT	%	RT	%	RT	%	
Tetrahydrofurfuryl chloride	3.38	2.10	-	-	-	-	-	-	-	-	-	-	Not reported
3-Hexanone	3.522	2.22	3.493	5.65	3.447	7.46	3.294	5.58	3.417	11.84	3.393	12.20	Antifungal activity [35]
2-Hexanone	3.653	3.99	3.622	7.59	3.571	8.22	3.395	7.11	3.535	13.64	3.506	14.48	Insecticide [36]
3-Hexanol	3.789	1.73	3.743	3.62	3.681	5.28	3.494	5.30	3.640	7.26	3.610	9.00	Antibacterial [37]
2-Hexanol	3.929	2.82	3.880	4.50	3.811	6.79	3.601	7.04	3.765	10.54	3.730	10.18	Biological control of pests [38]
Heptanoic acid	-	-	9.468	13.37	9.383	9.00	-	-	9.312	9.17	9.261	7.75	Marker of neuronal differentiation [39]
Hexanoic acid	-	-	-	-	-	-	7.415	0.83	-	-	-	-	Antifungal activity [40]
Octanoic acid	11.054	6.09	10.988	7.90	10.908	6.80	10.679	3.86	10.848	6.65	10.805	4.95	Antimicrobial activity [41]
2-Decenal, (E)-	12.107	6.05	-	-	-	-	-	-	-	-	-	-	Nematicidal activity [42]
2-Tridecenal, (E)-	-	-	12.073	4.16	13.540	1.93	-	-	-	-	-	-	Antioxidant activity [43]
Nonanoic acid	12.469	4.15	12.406	4.65	12.339	2.47	12.149	2.12	12.288	2.44	12.248	2.32	Antimicrobial activities [44]
Undecanal	12.713	3.69	-	-	-	-	-	-	-	-	-	-	Antifungal activity [45]
2-Undecenal	13.607	6.10	13.571	4.94	-	-	-	-	-	-	-	-	Mosquito-repellent activity [46]
Dodecanal	14.170	4.28	-	-	-	-	-	-	-	-	-	-	Antimicrobial activity [47]
2-Dodecenal	15.010	0.95	-	-	-	-	-	-	-	-	-	-	Nematicidal activity [42]
Tridecanal	15.530	2.95	-	-	-	-	-	-	-	-	-	-	Antimicrobial [48]
Tetradecanal	16.814	3.47	-	-	-	-	-	-	-	-	-	-	Antibacterial activity [49]
Dodecanoic acid	-	-	-	-	16.264	2.32	16.119	1.38	16.217	2.09	16.194	1.94	Antibacterial, antioxidant, and anti-apoptotic [50]
Pentadecanal-	18.031 20.283	4.27 1.06	17.995	2.14	-	-	-	-	-	-	-	-	Antibacterial activity [51]
Tetradecanoic acid	-	-	-	-	-	-	18.478	1.21	-	-	-	-	Antibacterial activity [52]
Hexadecanoic acid, methyl ester	20.351	5.66	20.314	8.77	20.288	5.99	20.216	3.26	20.264	4.89	20.254	4.88	Antibacterial activities [53]
n-Hexadecanoic acid	-	-	-	-	-	-	20.637	2.91	20.724	1.96	20.703	2.03	Antioxidant and antibacterial activities [54]
Octadecanoic acid, 2-propenyl ester	21.868	10.04	21.831	13.31	21.806 23.681	8.64 4.34	21.736	11.84	21.782	6.88	21.773 23.650	8.27 4.01	Antibacterial activity [55]
9-Octadecanoic acid, methyl ester	-	-	-	-	-	-	22.003	2.81	-	-	-	-	Antioxidant and anticancer [56]
2(3H)-Furanone, dihydro-5-tetradecyl-	22.325	9.16	-	-	-	-	-	-	-	-	-	-	Not reported
Octadecanoic acid, ethenyl ester	23.331	9.83	23.293	8.13	23.681	4.34	23.611	6.06	-	-	-	-	Antimicrobial [57]
Bis(2-ethylhexyl) phthalate	26.194	9.40	26.153	11.27	26.126	27.33	26.041	38.68	26.098	22.66	26.087	17.98	Antibacterial and antimutagenic activities [58,59]
Total compounds	20		14		13		15		12		12		

RT: retention time; %: area percentage; Zn: ZnONPs.

## Data Availability

Data are contained within this article.
